# Genomes of *Bacteroides ovatus*, *B. cellulosilyticus*, *B. uniformis*, *Phocaeicola vulgatus*, and *P. dorei* isolated from gut cavernous fistulous tract micropathologies in Crohn's disease

**DOI:** 10.1128/mra.01152-23

**Published:** 2024-02-27

**Authors:** Vaidhvi Singh, Gail West, Claudio Fiocchi, Fabio Cominelli, Caryn E. Good, Michael R. Jacobs, Alexander Rodriguez-Palacios

**Affiliations:** 1 Division of Gastroenterology and Liver Disease, Case Western Reserve University School of Medicine, Cleveland, Ohio, USA; 2 Digestive Health Research Institute, Case Western Reserve University School of Medicine, Cleveland, Ohio, USA; 3 Department of Inflammation and Immunity, Lerner Research Institute, The Cleveland Clinic Foundation, Cleveland, Ohio, USA; 4 University Hospitals Research and Education Institute, University Hospitals Cleveland Medical Center, Cleveland, Ohio, USA; 5 Department of Pathology, Case Western Reserve University, Cleveland, Ohio, USA; 6 Department of Pathology, University Hospitals Cleveland Medical Center, Cleveland, Ohio, USA; 7 Department of Molecular Biology and Microbiology, Case Western Reserve University School of Medicine, Cleveland, Ohio, USA; University of Maryland School of Medicine, USA

**Keywords:** *Bacteroidota*, *Bacteroides*, *Phocaeicola*, plasmid

## Abstract

Surgically removed bowels from Crohn's disease patients exhibit a novel form of micropathologies known as cavernous fistulous tract microlesions (CavFT), resembling fissures. We announce the genomes/plasmids and antimicrobial resistance genes of six CavFT bacterial isolates representing the *Bacteroidota* genera *Bacteroides* and *Phocaeicola*. Plasmids were identified in *Bacteroides cellulosilyticus* and *Phocaeicola vulgatus*.

## ANNOUNCEMENT

Improving our knowledge of microscopic pathologies that could explain Crohn's disease (CD) progression and complications, such as fistulas and fissures (1), is crucial (2, 3). We recently discovered small cavitating lesions (that differ from fistulas/fissures) resembling geological cavern formations within the gut wall of CD patients that required surgery to remove their inflamed bowel. Termed cavernous fistulous tract microlesions (CavFT) (4), intriguingly, such lesions harbor commensal bacteria. We previously reported the genome of two *Parabacteroides distasonis* strains (5, 6) isolated from two CD-CavFT patients with ileitis (5), and showed via genome-rearrangement analysis that the two isolates are uniquely clonal and probably transmissible in the community (6). Experimentally, CavFT *P. distasonis* induced cell toxicity/inflammation via the interaction of succinate with other CavFT bacteria (7, 8). We now report the genome of six other representative CavFT strains from the genera *Phocaeicola* and *Bacteroides* (Table 1).

**TABLE 1:** Genome and antimicrobial resistance genes for six representative species from the *Bacteroidota* phylum that have been isolated from cavitating/cavernous fistulous tract micropathologies (CavFT) in patients that undergo surgery for treatment of Crohn's disease

	*Bacteroides* spp. in CavFT lesions			*Phocaeicola* spp. in CavFT lesions		
Genome features strain designation	*B. uniformis* CavFT-hAR50	*B. ovatus* CavFT-hAR92	*B. cellulosilyticus* CavFT-hAR84	*P. vulgatus* CavFT-hAR107	*P. dorei* CavFT-hAR62	*P. vulgatus* CavFT-hAR11
*Reference genome* (genome size bp)	ATCC 8492 (4719097)	ATCC 8483 (6472384)	CL06T03C01 (7271073)	ATCC 8482 (5163189)	DSM 17855 (5625379)	ATCC 8482 (5163189)
*Our genomes:*	**CavFT-hAR50**	**CavFT-hAR92**	**CavFT-hAR84**	**CavFT-hAR107**	**CavFT-hAR62**	**CavFT-hAR11**
GenBank accession	JAWDEU000000000.1	JAWLLH000000000.1	JAWLLG000000000.1	JAWDHD000000000.1	JAWDEV000000000.1	JAWDET000000000.1
SRA	SRR26682829	SRR26681274	SRR26682789	SRR26682859	SRR26682886	SRR26682921
Sequencing depth (×)	224	281	461	331	310	239
Assembly size (bp)	4,949,941	7,091,243	7,105,331	5,751,554	5,743,612	5,067,155
Reads	15,291	27,671	41,459	23,408	23,285	16,925
Scaffolds^*a*^ (significantly more contigs in *P* vs. *B*; *P*-value = 0.0208)	2	3	7	14	13	9
N50 (bp)**^*b*^** (shorter in *P* vs. *B*; *P*-value = 0.0225)	4,637,482	4,467,655	3,827,627	598,191	1,105,313	4,133,441
L50	1	1	1	4	3	1
Plasmids (size bp) NCBI accession no.	None	None	1 (3,055 bp) JAWLLG010000005.1	None	None	1 (11,575 bp) JAWDET010000005.1
G + C content (**%**)	46.3817	42.09042	42.8222	42.854244	42.033897	41.903137
NCBI						
Genes (total)	4,147	5,613	5,343	4,905	4,757	4,309
CDSs (total)	4,069	5,504	5,260	4,790	4,654	4,203
Genes (coding)	3,992	5,404	5,170	4,614	4,519	4,069
CDSs (with protein)	3,992	5,404	5,170	4,614	4,519	4,069
Genes (RNA)	78	109	83	115	103	106
ncRNAs	2	3	3	2	2	2
tRNA	64	91	68	91	80	83
rRNA	12	15	12	22	21	21
Antibiotic Resistance BV-BRC [duplicated genes] (unique gene)^*c*^	24 [0] (gidB, Iso-tRNA, inhA, fabI, TetQ, EF-Tu, gyrA, MurA, GdpD, dxr, folP, S10p, Ddl, gyrB, S12p, folA, Dfr, OxyR, EF-G, kasA, rpoB, ErmB, rho, rpoC)	31 (rpoC, folP, gyrB, MurA, rho, EF-Tu, gyrA, S10p, EF-G, S12p, inhA, fabI, rpoB, TetQ, **MefA**, **CepA**, Iso-tRNA, dxr, **ErmG,** GdpD, kasA, OxyR, Ddl, **MsrD**, folA,Dfr, gidB, CfxA)	27 (EF-G, rpoC, inhA, fabI, EF-Tu, OxyR, GdpD, S12p, TetQ, Iso-tRNA, gyrB, dxr, folA, Dfr, kasA, rpoB, Ddl, S10p, gyrA, gidB, folP, rho, MurA)	29 (Ddl, MurA, MefEn2, EF-Tu, ErmF/Erm35, S10p, inhA, fabI, S12p, dxr, rho, ANT6-I, LnuAN2, gyrB, gyrA, Iso-tRNA, gidB, rpoB, folA, Dfr, rpoC, TetQ, kasA, EF-G, folP, TetX)	35 (folP, dxr, **NimB**, folA, Dfr, S12p, gyrB, OxyR, MurA, TetQ, kasA, Iso-tRNA, EF-G, TetX, gidB, ErmF/Erm35, ErmB, S10p, LnuAN2, MefEn2, gyrA, Ddl, rpoB, EF-Tu, inhA, fabI, rho, rpoC, GdpD, ANT6-I)	31 (Ddl, MefEn2, folP, EF-Tu, inhA, fabI, Iso-tRNA, gyrB, S10p, gyrA, ErmF/Erm35, TetX, gidB, rho, EF-G, folA, Dfr, MurA, LnuAN2, rpoB, CfxA, rpoC, S12p, dxr, kasA, ANT6-I, TetQ)

^*a*^ Significantly more contigs in *Phocaeicola* vs. *Bacteroides*; *P*-value = 0.0208, unpaired *T*-test.

^*b*^ Shorter N50 in *Phocaeicola* vs. *Bacteroides; P*-value = 0.0225, unpaired *T*-test.

^*c*^ The most common genes found in all six genomes (6/6) include gidB, Iso-tRNA, inhA, fabI, TetQ, EF-Tu, gyrA, MurA, dxr, folP, S10p, Ddl, gyrB, S12p, folA, Dfr, EF-G, kasA, rpoB, rho, and rpoC. Genes present in four out of the six lists (4/6) are GdpD and OxyR. Genes found in three out of the six lists (3/6) include MefEn2, ErmF/Erm35, ANT6-I, LnuAN2, and TetX. Genes found in two out of the six lists (2/6) include ErmB and CfxA. The least frequent genes, found in only one out of the six lists (1/6), are MefA, CepA, ErmG, MsrD, and NimB.

As previously described (4, 5), CavFT commensals were isolated under anaerobic conditions from debris harvested by microdissection of fresh surgically removed CD bowels. Debris was dissected under a microscope, plated, and purified anaerobically using pre-reduced 5% defibrinated sheep blood agar in tryptic soy medium (80% nitrogen; 10% hydrogen; 10% carbon dioxide; 37°C). Single colonies were sub-cultured on the same agar, then stored in 7% dimethyl sulfoxide pre-reduced brain heart infusion broth (9). Qiagen's MagAttract high-molecular-weight DNA kit was used to extract DNA (4, 5). Prior to genome sequencing, single colonies from purified isolates were speciated using matrix-assisted laser desorption ionization-time of flight mass spectrometry as previously described (5, 9, 10).

Long-range DNA genome sequencing was conducted using PacBio sequel II. To create the SMRTbell library, the SMRTbell template prep kit 1.0 was employed. Genomic DNA was sheared using the Covaris S220 Acoustic System. Subsequently, the sheared DNA was collected and purified using AMPure PB beads in accordance with the PacBio protocol. This involves the elimination of small fragments (<20 kb) using BluePippin. Prior to sequencing, the library underwent validation through an Agilent 2100 bioanalyzer (11). Sequencing was performed using a single-molecule real-time (SMRT) cell employing P6-C4 chemistry and PacBio sequel. The Flye 2.56 software was utilized for *de novo* assembly (12). Throughout the process, default parameters were applied in the software used, unless stated otherwise. For contextualization, BV-BRC (Bacterial and Viral Bioinformatics Resource Center) was used for automated annotation and overall comparison. The final annotation of these genomes for user accessibility was conducted by NCBI using the Prokaryotic Genome Annotation Pipeline, and the data provided are based solely on this annotation (Table 1). Processed using the same methods, the genomes are nearly complete for *Bacteroides* compared to *Phocaeicola*, with more contigs found in *Phocaeicola* than in *Bacteroides* (Table 1). Plasmids were identified in *Bacteroides cellulosilyticus* CavFT-hAR84 and *Phocaeicola vulgatus* CavFT-hAR11.

These genomes will further enhance our understanding of the potential pathogenic role of commensal *Bacteroides* in gut health (13, 14), particularly in the context of gut-wall micropathologies in CD and CavFT. Understanding genomic features that enable *Bacteroidota* as commensals or pathobionts, including their traits for antimicrobial resistance (Table 1), could be targeted for CD amelioration.

## Data Availability

The genome-sequenced data can be accessed on NCBI under the Bioproject accession number: PRJNA224116.
